# Controlling AGV While Docking Based on the Fuzzy Rule Inference System

**DOI:** 10.3390/s25196108

**Published:** 2025-10-03

**Authors:** Damian Grzechca, Łukasz Gola, Michał Grzebinoga, Adam Ziębiński, Krzysztof Paszek, Lukas Chruszczyk

**Affiliations:** 1 Department of Electronics, Electrical Engineering and Microelectronics, Silesian University of Technology, 44-100 Gliwice, Poland; lg300577@student.polsl.pl (Ł.G.); mg301705@student.polsl.pl (M.G.); lch@polsl.pl (L.C.); 2 Department of Distributed Systems and Informatic Devices, Silesian University of Technology, 44-100 Gliwice, Poland; adam.ziebinski@polsl.pl; 3 Department of Telecommunications and Teleinformatics, Silesian University of Technology, 44-100 Gliwice, Poland; krzysztof.paszek@polsl.pl

**Keywords:** Autonomous Guided Vehicles, fuzzy logic, docking algorithm, dead reckoning

## Abstract

Accurate docking of Autonomous Guided Vehicles (AGVs) is a critical requirement for efficient automated production systems in Industry 4.0, particularly for collaborative tasks with robotic arms that have a limited working range. This paper introduces a cost-effective software-upgrade solution to enhance the precision of the final docking phase without requiring new hardware. Our approach is based on a two-stage strategy: first, a switch from a global dead reckoning system to a local navigation scheme, is triggered near the docking station; second, a dedicated Takagi-Sugeno Fuzzy Logic Controller (FLC), guides the AGV to its final position with high accuracy. The core novelty of our FLC is its implementation as a gain-scheduling lookup table (LUT), which synthesizes critical state variables—heading error and distance error—from limited proximity sensor data, to robustly handle positional uncertainty and environmental variations. This method directly addresses the inadequacies of traditional odometry, whose cumulative errors become unacceptable at the critical docking point. For experimental validation, we assume the global navigation delivers the AGV to a general switching point, near the assembly station with an unknown true pose. We detail the design of the fuzzy controller and present experimental results that demonstrate a significant improvement, achieving repeatable docking accuracy within industrially acceptable tolerances.

## 1. Introduction

Autonomous Guided Vehicles (AGVs) [1] represent a significant application of autonomous technology, with wide utility across various sectors [2] such as manufacturing [3], agriculture, defense, and services [4]. AGVs have become a transformative force in the transportation industry [5], offering potential benefits in terms of safety, efficiency, and environmental impact. However, operating in complex environments presents challenges that necessitate the use of advanced algorithms for navigation, object detection, and decision-making.

Current systems enable AGVs to navigate safely and efficiently within production halls. Yet, achieving the necessary precision for docking operations is a critical challenge. Ensuring safety within industrial environments [6,7] and for the workers is crucial. Additionally, precise docking is vital, as emphasized in recent studies [8], for the successful execution of subsequent production processes [9], which often involve robots or collaborative robots [10].

The docking procedure [11] for an AGV presents unique challenges and requires specialized algorithms. Various sensor technologies have been applied: LiDAR for environment mapping [12], ultrasonic sensors for short-range distance measurement [13], radar for robust motion tracking [14], IMUs for orientation estimation [15], and wheel detectors [16]. These sensors facilitate environmental mapping and enhance positional accuracy [17], often through data fusion techniques [18] and the simultaneous localization and mapping (SLAM) method [19]. They also improve the precision [20] of local navigation [21] and autonomous driving of the AGV.

This research is motivated by a critical industrial constraint: the widespread deployment of existing AGV navigation systems, which are costly and logistically prohibitive to replace outright. Consequently, any enhancement must minimize hardware changes, ruling out solutions based on cameras or LiDAR [22]. The only industrially acceptable approach was a software update supplemented by low-cost proximity sensors. To address this, we developed a novel strategy where the AGV switches to a local navigation procedure upon approaching an assembly station (AS), leveraging a two-step fuzzy logic system to interpret the new sensor data [23]. This symbiosis of limited hardware change and intelligent software constitutes the core novelty of our work.

**Figure 1:**
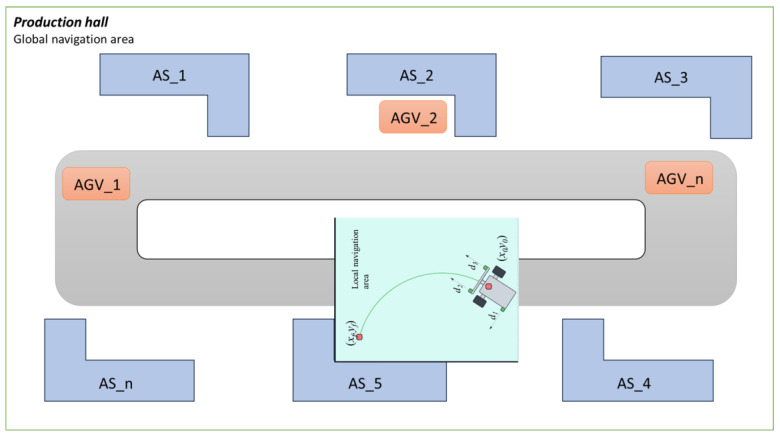
Production hall with exemplary assembly stations (global navigation area).

This research focuses on the analysis and implementation of an autonomous docking algorithm while approaches the AS. Docking, in this case, means stopping AGV at a particular position [24] with acceptable accuracy to cooperate with robotic arm. Therefore, an efficient local navigation algorithm is crucial for the effective operation of AGVs. The analysis covers the integration of sensor data [25], the interpretation of environmental cues, and the generation of control commands. The primary objective of the research was to develop a robust local docking algorithm that ensures a safe and efficient path [26] for the AGV to achieve accurate docking from its switching position [27] (Figure 1).

The novelty of the proposed solution arises from the navigation scheme, where switching between global and local navigation is mandatory, the use of low-cost range finders (time-of-flight, TOF), and a fuzzy logic controller with gain scheduler to navigate toward the docking point and stop at the final position. This solution can be implemented in hardware with only a software update. The approach aims to reach the docking point with accuracy better than that of the current dead reckoning algorithm. The efficiency of the developed method was validated through experiments conducted with an AGV system and compared to the traditional dead reckoning algorithm. These experiments were designed to assess the accuracy and reliability of the AGV’s positioning during docking maneuvers, which are critical for applications such as automated warehouses and manufacturing environments.

The paper is organized as follows: the second section presents the general concept of switching between global and local navigation, the third section describes the physical architecture of the AGV, the fourth section presents the Fuzzy Logic method developed for docking functionality, the fifth section presents currently implemented docking method, the sixth section details the docking experiments conducted and the last presents general conclusions.

### 1.1. Switching to Local Navigation–Docking Procedure

It is important to note that this problem of docking inaccuracy is not merely a function of the navigation system’s design, but also of its interaction with the operational environment. Even if a global navigation system’s theoretical accuracy and implementation cost are deemed acceptable, its final performance is compromised by unpredictable factors such as floor surface quality, load distribution, and tire wear. These variables introduce errors that are systematic yet non-repeatable, making them difficult to model and correct through calibration alone. Therefore, a solution is required that can adapt to these changing conditions in real-time, specifically during the critical final approach.

In a conventional implementation, the global navigation system often facilitates one-way transportation of loads by AGVs to Automated Storage (AS) throughout the production hall. However, this system allows the AGV to reach the designated AS only with limited precision. Since AGVs frequently transport loads of varying weights and sizes, which may also be loaded asymmetrically, this affects the pressure distribution on each wheel. Consequently, it can lead to errors in odometry, resulting in inaccurate measurements of the distance traveled by the AGV. This precision deficit becomes a critical failure point in systems where AGVs are integrated with collaborative robots (cobots) designed to assist human operators at the AS. The cobot’s operational range is finite and predicated on the AGV docking in an exact position and orientation. Precise alignment ensures the robotic arm has its full, optimal working envelope. Imprecise docking, however, severely constricts this range. This not only hinders functionality but fundamentally compromises safety—the cornerstone of human–robot interaction. A mis-docked AGV may violate the pre-defined collaborative workspace, forcing an unsafe intervention, a work stoppage, or a time-consuming and inefficient manual redocking procedure, nullifying the benefits of automation.

For this reason, when an AGV approaches a designated AS using global navigation (odometry), it becomes necessary to switch to local navigation. The local navigation system enables the AGV to travel to the docking point, for example, based on distance measurements to the shape of the specific AS. In the case of the AGV docking system studied as part of the CoBotAGV project, it was assumed that the side walls of the AS are L-shaped (see Figure 1).

**Figure 2:**
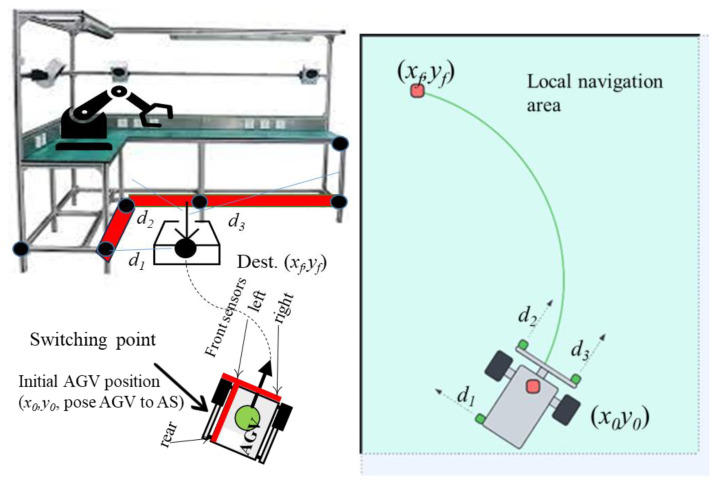
General concept of local navigation area.

Local navigation systems are specialized for fine-tuning the AGV’s movements within a confined space (see Figure 2). This involves handling intricate maneuvers and adjustments to achieve precise docking at the exact position required for effective operation. Local navigation typically employs detailed sensor data and sophisticated algorithms to manage these precise tasks.

While this research does not delve deeply into the intricacies of the transition between global (dead reckoning, odometry) and local navigation, it is essential to recognize that this switch is an important component in ensuring the AGV’s successful docking.

Given the critical importance of achieving precision in docking, we propose implementation of a fuzzy logic-based system. This approach is suggested to enhance the effectiveness of local navigation (Figure 2) by providing a more adaptive and responsive method for handling the uncertainties and dynamic conditions inherent in the docking process. The following sections will detail how the fuzzy logic controller integrates with the AGV system to improve docking accuracy and operational reliability.

Nevertheless, it must be emphasized that effective switching between global and local modes is essential to ensure the target stopping accuracy of the vehicle at the assembly station.

### 1.2. Docking Procedure

The docking procedure can be now described in steps:

1. **Initiation and Mode Switch:** The AGV navigates to a predefined switching point ($x_{0}$, $y_{0}$) using its global dead reckoning system. Upon reaching this point, the navigation mode seamlessly switches from global to local. This transition is subtle from a operational perspective but fundamental from a control standpoint, as it shifts reliance from odometry to real-time proximity sensor data for final precision maneuvering.
2. **Relative State Estimation:** In the local navigation mode, the AGV continuously acquires distance measurements ($d_{1}$), ($d_{2}$), and ($d_{3}$), from its onboard proximity sensors relative to the L-shaped assembly station. This raw data is processed and fused to estimate the vehicle’s precise relative state. This state is defined by:
  - Heading Error: the angular deviation from being parallel to the station.
  - Lateral Error: the displacement error perpendicular to the docking direction (e.g., distance to the left wall).
  - Longitudinal Error: the remaining distance to travel along the docking axis to reach the final point ($x_{f}$, $y_{f}$).
3. **Fuzzy Logic Control:** the estimated state variables (∆head, $∆d_{x}$, $∆d_{y}$) are fuzzified and fed into the Takagi-Sugeno Fuzzy Logic Controller (FLC). The FLC, operating on its rule base (implemented as a gain-scheduling lookup table), calculates the optimal PWM signals for the left and right wheels. This closed-loop control process continuously adjusts the AGV’s trajectory until the state errors are minimized.
4. **Docking Completion:** The procedure terminates successfully when the AGV’s calculated relative position resides within the acceptable tolerance window of the target destination (*x_f_*, *y_f_*), defined as $\left(x_{ref}\pm ∆x_{a},y_{ref}\pm ∆y_{a}\right)$. The AGV comes to a full stop, maintaining its position until the next command is received.

## 2. Fuzzy Logic Controller

**Figure 3:**
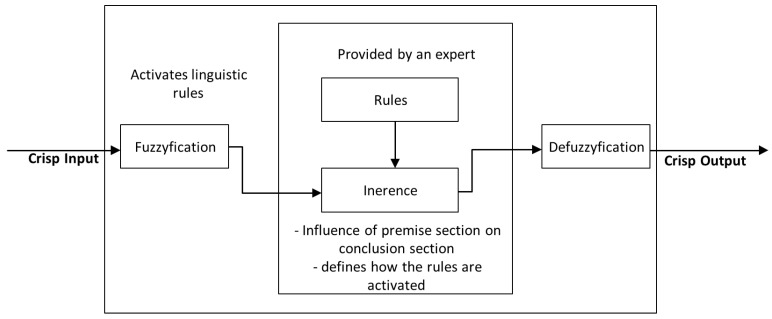
Structure of fuzzy logic controller (FLC).

Logic controllers based on fuzzy logic [28] are derived from fuzzy sets, a mathematical system that analyzes analog input values in terms of logical variables that take on continuous values between 0 and 1. The fuzzy inference system that controls the AGV is composed of blocks: fuzzification, inference system including rules and defuzzification that can be seen in Figure 3 [29,30].

The control system utilizes a fuzzy logic architecture. The crisp input values are the distances measured by the onboard proximity sensors. These crisp values are fuzzified using predefined linguistic variables.

The core of the local navigation system is a fuzzy logic controller (FLC) that translates sensor data into motor commands. The FLC was selected for its ability to handle imprecise inputs and nonlinear system dynamics without requiring complex mathematical models or computationally expensive processors, thus aligning with the project’s constraint of using existing hardware [31,32]. The controller’s operation follows a standard architecture:

1. Fuzzification: Crisp input values from the distance sensors are mapped into linguistic variables (e.g., “distance” is described by terms such as “very close,” “close,” “optimal,” “far,” and “very far”) using predefined membership functions. This process converts numerical sensor readings into fuzzy sets characterized by degrees of membership between 0 and 1.
2. Rule Inference: A set of IF-THEN rules uses these fuzzified inputs to make inferences about the appropriate motor response. The antecedent parts of these rules evaluate the fuzzy inputs, while the consequent parts define the output for each wheel’s PWM value.
3. Defuzzification: The aggregated fuzzy output sets from the rule evaluation are converted into a single, crisp value—the precise PWM duty cycle commanded to each wheel’s motor controller in case of Mamdami like system or a function for Takagi-Sugeno system [33,34].

Given the need for computational efficiency and a straightforward implementation in a dynamic industrial environment, the Takagi-Sugeno (Type 1) Fuzzy Inference System was adopted for this project. In the Takagi-Sugeno model, the consequent part of the rules is typically a linear function or a constant, which simplifies the defuzzification process to a weighted average, making it less computationally intensive than the Mamdani model. This efficiency is critical for achieving rapid, real-time control responses on low-cost hardware during the precise docking maneuver.

### 2.1. Preliminary Assumptions

A predefined accuracy for reaching destination point $\left(x_{f},y_{f}\right)$ can be defined as follows:

(1) $$\left(x_{f},y_{f}\right)=\left(x_{ref}\pm ∆x_{a},y_{ref}\pm ∆y_{a}\right)$$

where *x_f_* and *y_f_* are coordinates of the docking point, *x_ref_* and *y_ref_* are final coordinates of the AGV (after stopping), $∆x_{a}$ and $∆y_{a}$ are predefined acceptable errors. These values are determined based on the specific requirements of the collaborative task (e.g., the tolerance required for the robotic arm to successfully engage with the load).

The transition from the global navigation system (dead reckoning) to the local fuzzy logic controller (FLC) is triggered at a specific switching point $\left(x_{0},y_{0}\right)$. This point is defined relative to the target assembly station. Upon switching, the coordinate frame of reference for the AGV becomes local, with the origin in its corner (L-shaped). Consequently, the AGV’s position during the entire FLC-controlled docking phase is denoted and tracked relative to this local frame, ensuring the controller’s rules are applied consistently regardless of the AGV’s global position in the facility.

### 2.2. Fuzzy Input and Output Variables

Fuzzy controller requires linguistic variables at the input and output, so the crisp values are compared to linguistic values that are described by membership functions, e.g., “distance is close” or “distance is OK”, where “distance” is linguistic variable, “close” and “OK” are linguistic values.

**Figure 4:**
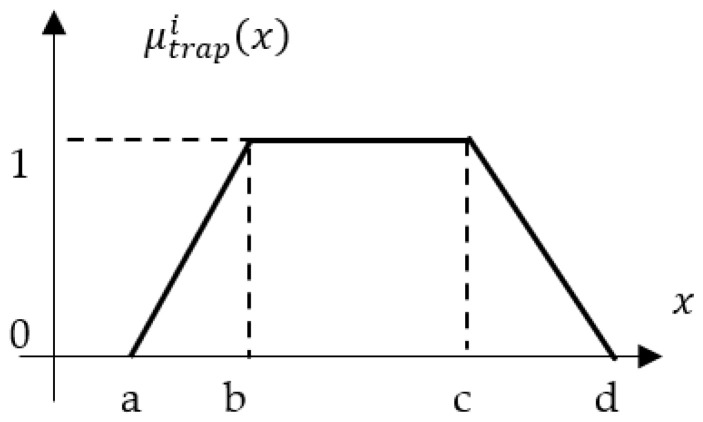
Trapezoidal membership function.

The inputs to the fuzzy logic controller (FLC) are the crisp distance measurements acquired from the three proximity sensors, denoted as *d*_1_, *d*_2_, and *d*_3_. Each input variable is defined within its own universe of discourse, a numerical range determined by the operational limits of the corresponding sensor. For example, the variable “*distance d*_1_” has a universe of discourse from 2 cm to 2 m, representing its minimum and maximum reliable sensing range. Each linguistic variable (e.g., “*distance d*_1_”) is characterized by a set of linguistic values. For this implementation, the set {FAR, CLOSE, OK} was chosen to describe the AGV’s relative position to the assembly station. These values are mathematically represented by trapezoidal membership functions, as illustrated in Figure 4. The trapezoidal membership function is defined by four parameters *a*, *b*, *c*, and *d* that shapes its profile. The degree of membership $\mu_{Trapezoid}(x)$ for a given crisp input value *x* is calculated as:

(2) $$\mu_{Trapezoid}\left(x;a,b,c,d\right)=\max\left(\min\left(\frac{x-a}{b-a},1,\frac{d-x}{d-c}\right),0\right)$$

where *a*, *b*, *c*, *d* are the constants that define the start of the ramp-up, the start of the core, the end of the core, and the end of the ramp-down of the trapezoid, respectively, with a≤b≤c≤d. Constants are usually tuned experimentally to optimize the AGV’s docking behavior, balancing responsiveness and stability.

Given the limited number of physical sensors, two additional linguistic variables were synthesized to provide the fuzzy controller with the necessary state information for precise docking: Alignment (heading error) and Distance Error.

Alignment (Heading Error): This linguistic variable represents the angular deviation (∆head) of the AGV with respect to the L-shaped station. It is derived indirectly from the history of readings from the left-side distance sensor *d*_1_, under the principle that a changing distance to a parallel surface indicates a rotational drift. To calculate this robustly and reduce noise, a moving average filter with an adjustable window size (e.g., windows size 5) is applied to the *d*_1_ sensor readings. The heading error at the time *i* is then defined:

(3) $$∆head=\left({\bar{d}}_{1}^{i}-{\bar{d}}_{1}^{i-1}\right)$$

where ${\bar{d}}_{1}^{i}$ is a moving average at the current time step, ${\bar{d}}_{1}^{i-1}$ is a moving average at the previous time step.

This error term is fuzzified into the linguistic variable “Heading Error” with the set of values {NEGATIVE, OK, POSITIVE}. A positive value indicates the AGV is rotating away from the station, while a negative value indicates it is rotating towards it.

The second linguistic variable controls the stopping point and it is labels “distance error”. It is a distance to the front of the L-shaped station. Having two distance sensors on a front bumper, the longitudinal distance error can be formulated:

(4) $$∆d_{y}=y_{f}-\left(\frac{d_{3}+d_{2}}{2}\right)$$

The third linguistic variable controls the distance between AGV and wall, is called a lateral distance error:

(5) $$∆d_{x}=x_{f}-d_{1}$$

Linguistic variables “distance error” is fuzzified using linguistic values {NEGATIVE, OK, POSITIVE}. All values are defined by trapezoidal membership functions.

The output of the Takagi-Sugeno fuzzy inference system is designed around two conceptual linguistic variables that govern the AGV’s movement in a form of functions. However, to better understand the concept lets introduce linguistic variables:

1. “Movement” determines the translational command, with the linguistic values {BACKWARD, STOP, FORWARD}.
2. “Rotation” determines the rotational command, with the linguistic values {RIGHT, NO_TURN, LEFT}.

Both variables has impact on coefficients $a_{R}$, $a_{L}$, $b_{R}$, and $b_{L}$. The output crisp values are *PWM_L_* and *PWM_R_* for the left and right wheels, respectively. In the Takagi-Sugeno model used in this work, the consequent part of each fuzzy IF-THEN rule is a linear function of the input memberships and a Look Up Table (LUT). The final crisp output for each wheel is calculated as a weighted average of all rule consequents, governed by the following equations (control law):

(6) $$\begin{array}{c} PWM_{L}={PWM}_{min}+a_{L}\cdot \mu \left(∆head\right)+b_{L}\cdot \mu \left(∆d_{x,y}\right)\\ PWM_{R}={PWM}_{min}+a_{R}\cdot \mu \left(∆head\right)+b_{R}\cdot \mu \left(∆d_{x,y}\right) \end{array}$$

where $PWM_{L}$, $PWM_{R}$ are functions output directly controls left and right motors of AGV, $PWM_{min}$ is a constant representing the minimum PWM value required to initiate wheel rotation, overcoming static friction, $\mu \left(∆head\right)$, $\mu \left(∆d_{x,y}\right)$ are the aggregated membership values (the fuzzy degrees of truth) for the respective input variables, $a_{R}$, $a_{L}$, $b_{R}$, $b_{L}$ are constant gains tuned experimentally.

The gains determine the influence of each input variable on the motor output and are dependent on factors such as the PWM output range, the AGV’s mass, and motor characteristics. Crucially, the values of these coefficients are dynamically influenced by the current stage of the docking procedure, effectively implementing the conceptual “Movement” and “Rotation” commands.

The inference process for the conceptual output variables “Movement” and “Rotation” is optimized for real-time performance on low-cost hardware. Rather than calculating outputs dynamically, the controller references a pre-defined lookup table (LUT) with predefined coefficients $a_{R}$, $a_{L}$, $b_{R}$, $b_{L}$ corresponding to every possible combination of the discretized fuzzy input states: heading error, longitudinal error and lateral error.

The design of this LUT embodies the complete set of fuzzy IF-THEN rules.

Each cell in the 3D LUT contains a gain set or parameter set: $a_{R}$, $a_{L}$, $b_{R}$, $b_{L}$ for that specific operational state. Example: The cell indexed by (HEADING = OK, DX = OK, DY = NEGATIVE) might contain gains ($a_{R}=0$, $a_{L}=0$, $b_{R}=-30$, $b_{L}=-30$), which would generate a BACKWARD command in the output functions, because the b coefficients are negative and multiply the distance error.

Each entry in the table represents the consequent of a rule, where the indices are the fuzzy values of the inputs (e.g., Heading_Error = NEGATIVE, Distance_Error = OK), and the table cell contains the resulting command pair (Movement, Rotation) (e.g., FORWARD, LEFT) The choice of these linear functions as consequents is a key feature of the Sugeno model, simplifying the defuzzification step to a simple weighted average and reducing computational overhead, which is crucial for real-time performance on low-cost hardware. The procedure can be formulated in three steps:

1. State Evaluation: Fuzzify inputs to get indices.
2. Gain Scheduling: Use indices to fetch gains ($a_{R}$, $a_{L}$, $b_{R}$, $b_{L}$) from LUT.
3. Control Calculation: Compute *PWM_L_* and *PWM_R_* using the control law and the fetched gains.

### 2.3. LUT as a Gain Scheduler

It retains the speed of a LUT-based approach. The potentially complex calculation of the gains is performed offline; the online controller just performs a fast table lookup and a simple multiply-accumulate operation.

As mentioned earlier the Inputs (LUT Indices):

1. **Heading Error** ∆head**:** {NEGATIVE, OK, POSITIVE}.
2. **Longitudinal Error** $∆d_{y}$**:** {NEGATIVE, OK, POSITIVE} (Distance to front wall).
3. **Lateral Error** $∆d_{x}$**:** {NEGATIVE, OK, POSITIVE} (Distance to left wall).
4. **Outputs (LUT Values):** Each cell in the 3D LUT contains a gain set or parameter set: ($a_{R}$, $a_{L}$, $b_{R}$, $b_{L}$) for that specific operational state. To make it readable, the 3D LUT is described in the following 3 tables for $∆d_{y}=$ NEGATIVE (Table 1), $∆d_{y}=$ OK (Table 2), and $∆d_{y}=$ POSITIVE (Table 3).

To make it even more understandable, below is the real world interpretation of the rules:

1. **Too Close and Drifting Left** (NEGATIVE, NEGATIVE): (STOP, RIGHT)
  - Logic: Emergency stop to avoid collision. Turn right to correct the leftward drift and realign parallel to the left wall.
2. **Too Close and Parallel** (OK, NEGATIVE): (BACKWARD, NO_TURN)
  - Logic: Reverse straight back to reach the target distance. Since parallel, no turning is needed.
3. **Too Close and Drifting Right** (POSITIVE, NEGATIVE): (STOP, LEFT)
  - Logic: Emergency stop. Turn left to correct the rightward drift and realign.
4. **Target Distance and Drifting Left** (NEGATIVE, OK): (FORWARD, RIGHT)
  - Logic: Distance is good but not aligned. Move forward while arcing right to gradually correct heading without losing alignment.
5. **Target Distance and Parallel** (OK, OK): (STOP, NO_TURN)
  - Logic: Docking Complete. Perfectly aligned at the correct distance. Stop and hold position.
6. **Target Distance and Drifting Right** (POSITIVE, OK): (FORWARD, LEFT)
  - Logic: Mirror of the above. Move forward while arcing left to correct heading.
7. **Too Far and Drifting Left** (NEGATIVE, POSITIVE): (FORWARD, RIGHT)
  - Logic: Need to get closer. Move forward while turning right to simultaneously reduce distance and correct heading.
8. **Too Far and Parallel** (OK, POSITIVE): (FORWARD, NO_TURN)
  - Logic: Simply move straight forward to reach the target distance.
9. **Too Far and Drifting Right** (POSITIVE, POSITIVE): (FORWARD, LEFT)
  - Logic: Need to get closer. Move forward while turning left to correct the drift.

The LUT defines how the AGV should behave in every possible scenario, e.g., tuning according to any high level strategy: “aggressive” for far away, “cautious” when close to the target. The control law executes that strategy smoothly. This separation makes the system easier to design, tune, and debug.

## 3. Implementation of the Fuzzy Controller

**Figure 5:**
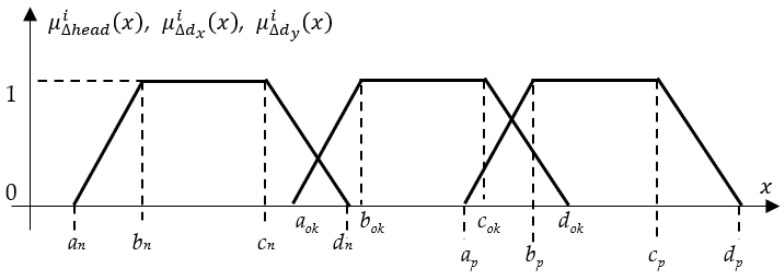
Distances Membership Functions.

For each input linguistic value, three fuzzy membership functions are defined (Figure 5): positive, ok, negative. The fuzzy controller was implemented using membership coefficients listed in Table 4. These fuzzy sets allow the system to interpret sensor readings and output appropriate motor speeds in a way that accounts for uncertainty and gradations.

### 3.1. AGV Platform Overview

**Figure 6:**
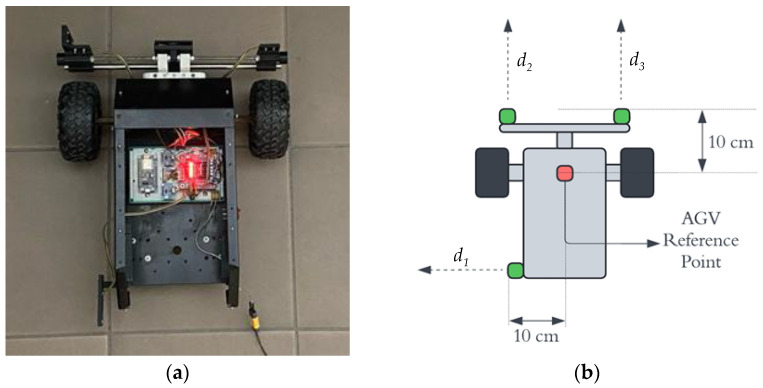
Top view of the AGV (**a**); visualization of parameters (**b**).

The proposed docking system was validated using a custom-built AGV platform. The AGV has a wheelbase (pitch) of 300 mm and a maximum translational velocity of 0.23 m/s. The core of the vehicle is a Raspberry Pi 4 microcontroller, which executes the high-level navigation algorithms, including the global dead reckoning and the proposed fuzzy logic controller. The AGV features a differential drive configuration, powered by two Brushless DC (BLDC) motors with integrated encoders for odometry feedback used in the global navigation phase. Each motor is driven by an H-bridge motor driver circuit, which allows for bi-directional speed control via Pulse Width Modulation (PWM) signals generated by the Raspberry Pi. This setup is illustrated in Figure 6.

For perception in the local navigation phase, the AGV is equipped with a suite of low-cost infrared proximity sensors. These include: a left-facing sensor $d_{1}$ for measuring lateral distance to the assembly station; two front-facing sensors ($d_{2}$, $d_{3}$) for measuring longitudinal distance and calculating lateral offset. The sensors used were VL53L0X Time-of-Flight (ToF) laser ranging modules. Their stated accuracy is ± 3% of the measured distance, with a maximum range of 2 m. This choice was done due to their low cost, small form factor, and digital interface.

### 3.2. Actuator Characterization and PWM Calibration

A critical prerequisite for precise control was the empirical characterization of the motor response. A series of tests was conducted to determine the operational range of the PWM control signals. Minimum PWM ($PWM_{min}$): The minimum value required to overcome static friction and initiate movement was determined to be $PWM_{min}=40$ (on a 0–255 scale). This ensures reliable motor engagement and was directly applied in the fuzzy control law. Maximum PWM and Velocity Calibration: The maximum PWM value was set to $PWM_{max}=115$ to prevent motor saturation. The AGV’s velocity was calibrated by measuring the time required to traverse a fixed distance of three meters, this yielded a maximum linear velocity of 230 mm/s at $PWM_{max}$.

This calibration defined the effective control range for the fuzzy controller, ensuring its output commands map to predictable and safe vehicle kinematics.

### 3.3. Reference Points and Performance Metrics

To ensure a reliable assessment of docking performance, reference points were established. The global reference point was defined as the corner (0, 0) of the L-shaped assembly station. The AGV’s performance was measured by calculating the Euclidean error between its final position and this target. A successful docking was defined as a final position within the predefined industrially acceptable tolerances:

(7) $$\left(x_{f},y_{f}\right)=\left(x_{ref}\pm ∆x_{a},y_{ref}\pm ∆y_{a}\right)$$

Furthermore, the switching point ($x_{0}$*,* $y_{0}$) served as a critical reference for initiating the local navigation procedure. For each experimental trial, the global navigation system was tasked with delivering the AGV to this predefined starting point for the fuzzy controller, ensuring a consistent initial condition for evaluating the local algorithm’s performance across multiple runs.

This framework of reference points provided a consistent basis for comparing experimental results, enabling an accurate statistical assessment of the precision and repeatability of the proposed docking method.

### 3.4. Testing Setup

**Figure 7:**
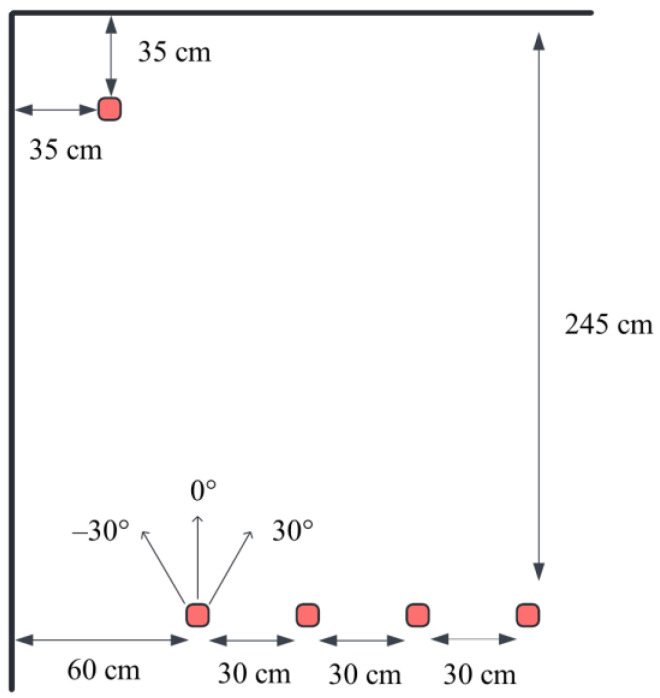
Visualization of the test procedure.

The proposed fuzzy logic docking controller was implemented in a Python 3.12 script and evaluated on the AGV platform described in Section 3.1. The primary performance criterion was docking accuracy—specifically, the final positional and angular error from the local navigation phase—rather than travel time or speed. The testing environment and initial conditions for the local navigation phase were defined under the following assumptions:

The target docking station was modeled as an L-shaped corner with dimensions of 350 mm, 350 mm (see Figure 7). The reference point for the final position was defined as the corner (0, 0). The switching points—where global navigation ends and the proposed local fuzzy controller takes over—were defined at four distinct starting positions (SP), given by their coordinates relative to the target corner (0, 0):

1. SP1 = (600 mm, 2450 mm)
2. SP2 = (900 mm, 2450 mm)
3. SP3 = (1200 mm, 2450 mm)
4. SP4 = (1500 mm, 2450 mm)

At each switching point, the local navigation maneuver began from three initial orientations: −30°, 0°, and + 30°, with the goal of achieving a final heading of 0° (perfect alignment).

### 3.5. Experimental Procedure

This design of experiment resulted in a total of 12 unique test scenarios (4 switching points × 3 initial angles). Each scenario was executed three times to assess the repeatability and reliability of the fuzzy controller, yielding a total of 36 experimental runs. The AGV’s final docked position and orientation were recorded based on its onboard sensor readings for the closed-loop operation, with validation provided by an external high-accuracy measurement system to ensure ground truth accuracy.

This comprehensive testing matrix was designed to rigorously evaluate the local controller’s performance from various approach vectors, challenging its ability to compensate for both significant positional offsets and angular misalignment after the handover from global navigation.

### 3.6. Results

Following the experimental procedure outlined in previous section, a full series of tests was conducted. The collected data was systematically analyzed to evaluate the performance of the proposed fuzzy logic controller against the built-in dead reckoning method, which served as the baseline. All tests were executed under controlled conditions to ensure consistency and reproducibility across all trials. The detailed results for each individual test across all starting positions and initial angles are presented in Table 5 (Proposed Fuzzy Logic Controller) and Table 6 (Baseline Dead Reckoning). These tables provide a complete record of the final positional *x_ref_*, *y_ref_* and angular *a_end_* state for every trial, along with the calculated errors.

A summary of the experimental results for all test scenarios is provided in Table 7 (Proposed Fuzzy Logic Controller) and Table 8 (Baseline Dead Reckoning). These tables catalog the final docking position $x_{ref}$, $y_{ref}$, orientation $a_{end}$, and corresponding errors for each individual trial, organized by starting position and initial angle.

**Figure 8:**
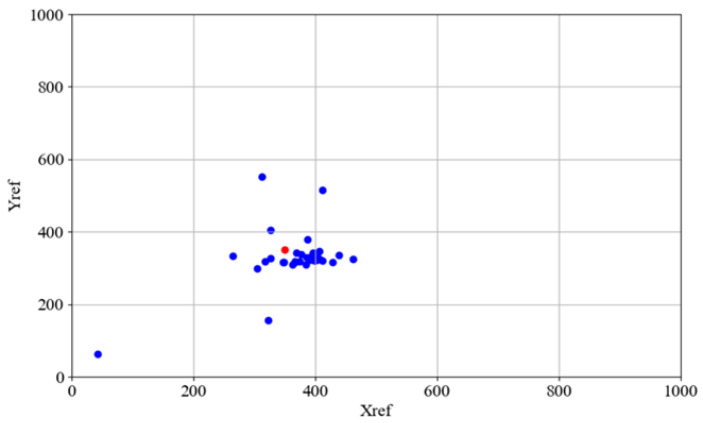
Docking position for fuzzy controller–coordinates of AGV docking marked by blue dots, the docking point marked by red dot.

**Figure 9:**
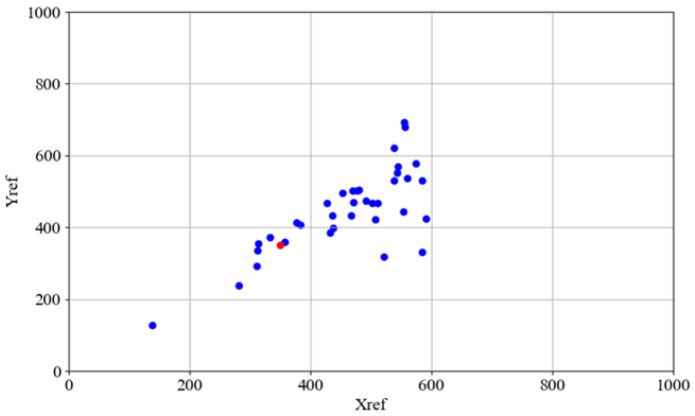
Docking positions for dead reckoning algorithm–coordinates of AGV docking marked by blue dots, the docking point ($x_{f}$, $y_{f}$) marked by red dot.

The aggregate docking performance is visually summarized in Figure 8 (Proposed Fuzzy Logic Controller) and Figure 9 (Baseline Dead Reckoning Algorithm). In both figures, the target destination point is indicated in red, while the final positions of the AGV from all completed trials are plotted in blue. The results demonstrate a significant improvement in both precision and repeatability achieved by the fuzzy logic controller. Figure 8 shows a cluster of blue points around the red target, confirming the system’s accuracy and consistency. Figure 9 reveals a widespread and unpredictable scatter of points for the dead reckoning system, highlighting its fundamental inadequacy for the precision docking task.

The overall docking performance for all experimental trials is quantitatively summarized in Table 9, which presents the calculated final positions and orientations for both the proposed fuzzy logic controller and the baseline dead reckoning method. The results demonstrate a definitive and significant general improvement in docking precision achieved by the fuzzy controller. Statistical analysis confirms that only the fuzzy controller consistently achieved performance within the predefined industrially acceptable tolerances. The average final displacement error was reduced to within the required range, and the final angular rotation was maintained at an acceptable level across all starting points and initial conditions. In contrast, the dead reckoning system exhibited substantial and unpredictable errors in both position and angle, failing to meet the required specifications in any of the conducted trials.

**Figure 10:**
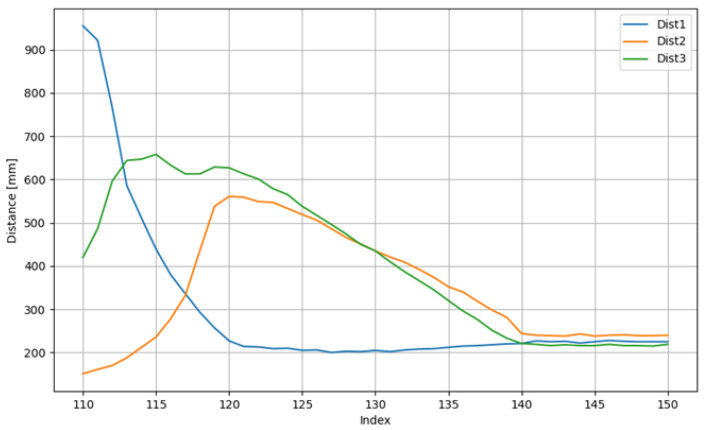
Exemplary distances to walls from corresponding sensors for a trial.

**Figure 11:**
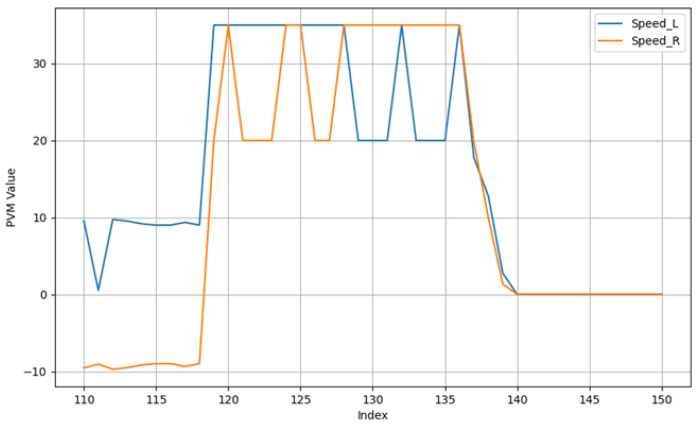
PWM Output functions for both wheels for a trial.

To elucidate the internal decision-making process of the fuzzy logic controller during a typical docking maneuver, the system’s inputs and outputs for a single exemplary trial are presented. Figure 10 illustrates the real-time distance measurements from the three proximity sensors (*d*_1_, *d*_2_, *d*_3_) as the AGV approaches the station. The corresponding motor control signals (PWM for the left and right wheels), generated by the fuzzy inference system, are shown in Figure 11.

The sensor data in Figure 10 demonstrates the system’s response to the changing environment, notably the moment the front sensors (*d*_2_, *d*_3_) detect the approaching station wall. Figure 11 reveals how the controller translates this sensory information into action: the PWM values adjust dynamically, initially to correct the vehicle’s heading and trajectory, and subsequently to reduce speed for a smooth and precise final approach until both motors are commanded to stop (PWM = 0) upon successful docking.

The most precise and repeatable results were obtained with a starting angle of 30° at starting positions SP1 and SP3. As detailed in Table 5 and Table 6, these configurations yielded the lowest absolute error and smallest standard deviation across all trials. For instance at SP1 with a 30° angle, the mean Euclidean error was minimized, with component errors of 17 mm (x-axis, SD = 15 mm) and 36 mm (y-axis, SD = 3 mm). Similarly, at SP3 with a 30° angle, the system demonstrated high repeatability, with standard deviations of 9 mm and 46 mm for the x and y coordinates, respectively. Relatively low errors were also observed with a **0°** starting angle at positions **SP2** and **SP4**. For **SP2** at **0°**, the mean error was **73 mm** (x-axis, SD = 29 mm) and **23 mm** (y-axis, SD = 6 mm), indicating consistent performance despite the increased distance.

In summary, the proposed fuzzy logic controller consistently achieved lower errors than the dead reckoning baseline. The overall mean positional error for the fuzzy controller was **less than 60 mm**, while the dead reckoning system exhibited errors frequently exceeding **200 mm**. This represents an average improvement in accuracy of over **70%**.

The fuzzy logic controller maintained a mean angular deviation below **9 degrees** for all scenarios, with most under 5 degrees. Whereas, the dead reckoning system suffered from angular errors, often between **60 and 80 degrees**, rendering the AGV completely misaligned at the end of its maneuver.

A practical finding of this research is that the precision of the final docking maneuver is influenced by the conditions at the switching point. The analysis demonstrates that to maximize docking accuracy, the switching point should not be chosen arbitrarily. Based on the experimental results, the following operational guideline should be considered:

The AGV should be oriented with a positive initial heading angle—approximately 30°—relative to the target station when initiating the local navigation phase. This specific approach vector consistently yielded the highest precision and repeatability across multiple starting positions (notably SP1 and SP3), as evidenced by the lowest mean errors and standard deviations reported in Table 6. This finding suggests that the rule base of the developed fuzzy logic controller is particularly effective at compensating for deviations and converging to the target when presented with this specific initial geometric relationship. Therefore, a minor pre-orientation maneuver by the global navigation system to achieve this 30° approach angle at the switching point is recommended to optimize the overall performance and reliability of the docking process.

Our fuzzy rule base and Look-Up Table (LUT) are designed to prioritize lateral alignment first (correcting heading error to become parallel to the station) and longitudinal approach second (closing the distance). A 30° angle presents a structured initial condition that perfectly suits this strategy. The rules for “HEADING = POSITIVE” (e.g., “turn LEFT”) are immediately and strongly activated, efficiently swinging the AGV into alignment.

## 4. Conclusions

This study addressed the critical challenge of achieving precise AGV docking in industrial environments. Although dead reckoning was a project requirement and can theoretically achieve ± 25 mm accuracy for an unloaded AGV, it proved unreliable in practice.

The proposed solution—a hybrid navigation strategy—was validated by the results. This approach requires an AGV to switch from global navigation (dead reckoning) to a local navigation scheme upon reaching the designated docking area. The local navigation is proposed with the use of a fuzzy logic controller supported by low-cost distance sensors. This architecture combines the broad-range positioning of dead reckoning with precise local correction, overcoming the latter’s fundamental limitations.

The implemented fuzzy logic controller with a gain scheduler demonstrated performance, maintaining angular alignment typically within 10 degrees—a capability good enough for docking: ± 25 mm precision target with a sufficient operational margin. Overall, the fuzzy logic–based method offers a robust, low-cost, and easily implementable software solution for Industry 4.0 environments, ensuring docking accuracy despite uncertainties in, e.g., initial position.

While this study focused on a comparison against the deployed dead reckoning baseline, a valuable direction for future work would be a comprehensive benchmarking study comparing the proposed fuzzy gain-scheduling controller against other advanced methods (e.g., PID, MPC) in a more open experimental setting, independent of specific industrial hardware constraints. Furthermore, the performance of the fuzzy controller could be further refined by employing data-driven optimization techniques, such as clustering or adaptive neuro-fuzzy inference systems (ANFIS), to automatically tune the membership functions and rule base.

## Figures and Tables

**Table 1:** Too Close to Front Wall (Longitudinal Error $∆d_{y}=$ NEGATIVE).

Layer 1 of 3	$∆d_{x}$ Is NEGATIVE	$∆d_{x}$ Is OK	$∆d_{x}$ Is POSITIVE
∆head is NEGATIVE	BACKWARD, RIGHT (10, −10, −30, −30)	BACKWARD, RIGHT (15, −15, −25, −25)	BACKWARD, RIGHT (20, −20, −20, −20)
∆head is OK	BACKWARD, NO_TURN (0, 0, −40, −40)	BACKWARD, NO_TURN (0, 0, −35, −35)	BACKWARD, NO_TURN (0, 0, −30, −30)
∆head is POSITIVE	BACKWARD, LEFT (−10, 10, −30, −30)	BACKWARD, LEFT (−15, 15, −25, −25)	BACKWARD, LEFT (−20, 20, −20, −20)

**Table 2:** At Target Distance (Longitudinal Error $∆d_{y}=$ OK).

Layer 2 of 3	$∆d_{x}$ Is NEGATIVE	$∆d_{x}$ Is OK	$∆d_{x}$ Is POSITIVE
∆head is NEGATIVE	STOP, RIGHT (20, −20, 0, 0)	STOP, RIGHT (30, −30, 0, 0)	SLOW_FWS, RIGHT (25, −25, 10, 10)
∆head is OK	STOP, NO_TURN (0, 0, 0, 0)	STOP, NO_TURN (0, 0, 0, 0)	SLOW_FWD, NO_TURN (0, 0, 15, 15)
∆head is POSITIVE	STOP, LEFT (−20, 20, 0, 0)	STOP, LEFT (−30, 30, 0, 0)	SLOW_FWD, LEFT (−25, 25, 10, 10)

**Table 3:** Too Far From Front Wall (Longitudinal Error $∆d_{y}=$ POSITIVE).

Layer 3 of 3	$∆d_{x}$ Is NEGATIVE	$∆d_{x}$ Is OK	$∆d_{x}$ Is POSITIVE
∆head is NEGATIVE	FORWARD, RIGHT (15, −15, 25, 25)	FORWARD, RIGHT (10, −10, 30, 30)	FORWARD, RIGHT (25, −25, 35, 35)
∆head is OK	FORWARD, NO_TURN (0, 0, 20, 20)	FORWARD, NO_TURN (0, 0, 40, 40)	FORWARD, NO_TURN (0, 0, 45, 45)
∆head is POSITIVE	FORWARD, LEFT (−15, 15, 25, 25)	FORWARD, LEFT (−10, 10, 30, 30)	FORWARD, LEFT (−25, 25, 35, 35)

**Table 4:** Coefficients for trapezoidal membership functions.

Membership Function	Value = NEGATIVE	Value = OK	Value = POSITIVE
$\mu_{∆d_{y}}^{i}\left(x\right)$	$a_{n}=-500$ $b_{n}=-500$ $c_{n}=-25$ $d_{n}=0$	$a_{ok}=-500$ $b_{ok}=-25$ $c_{ok}=25$ $d_{ok}=500$	$a_{p}=0$ $b_{p}=25$ $c_{p}=500$ $d_{p}=500$
$\mu_{∆d_{x}}^{i}\left(x\right)$	$a_{n}=-500$ $b_{n}=-500$ $c_{n}=-25$ $d_{n}=0$	$a_{ok}=-500$ $b_{ok}=-25$ $c_{ok}=25$ $d_{ok}=500$	$a_{p}=0$ $b_{p}=25$ $c_{p}=500$ $d_{p}=500$
$\mu_{∆head}^{i}\left(x\right)$	$a_{n}=-500$ $b_{n}=-500$ $c_{n}=-20$ $d_{n}=0$	$a_{ok}=-500$ $b_{ok}=-15$ $c_{ok}=15$ $d_{ok}=500$	$a_{p}=0$ $b_{p}=20$ $c_{p}=500$ $d_{p}=500$

**Table 5:** Detailed results for Fuzzy Logic Algorithm.

Starting Position (*x*_0_*, y*_0_) [mm, mm]	*a_start_* [deg]	Attempt	*x_ref_* [mm]	*y_ref_* [mm]	*a_end_* [deg]	*Error x_ref_* [mm]	*Error y_ref_* [mm]	*Error a_end_* [deg]
SP1 (600, 2450)	−30	1	363	310	−6	−13	40	6
		2	428	317	−3	−78	33	3
		3	396	342	−11	−46	8	11
	0	1	397	341	−6	−47	10	6
		2	347	315	−4	3	35	4
		3	392	323	−6	−42	27	6
	30	1	366	319	−3	−16	32	3
		2	348	315	−2	2	35	2
		3	384	310	−1	−34	40	1
SP2 (900, 2450)	−30	1	265	333	−11	85	17	11
		2	220	323	−9	130	27	9
		3	406	347	−13	−56	3	13
	0	1	462	325	−7	−112	25	7
		2	411	321	−9	−61	30	9
		3	395	336	−8	−45	15	8
	30	1	326	327	−4	24	24	4
		2	323	155	−2	27	195	2
		3	387	379	−3	−37	−29	3
SP3 (1200, 2450)	−30	1	400	335	−10	−50	16	10
		2	439	336	−6	−89	15	6
		3	369	342	−11	−19	8	11
	0	1	399	321	−6	−49	29	6
		2	374	318	−10	−24	33	10
		3	402	322	−5	−52	28	5
	30	1	326	405	2	24	−55	−2
		2	317	319	8	33	31	−8
		3	304	299	−1	46	52	1
SP4 (1500, 2450)	−30	1	377	337	−12	−27	13	12
		2	405	327	−8	−55	23	8
		3	412	516	−8	−62	−166	8
	0	1	367	317	−3	−17	34	3
		2	386	329	−5	−36	21	5
		3	407	325	−4	−57	26	4
	30	1	310	334	1	40	16	−1
		2	250	323	−2	100	27	2
		3	312	552	3	38	−202	−3

**Table 6:** Detailed results for Dead Reckoning Algorithm.

Starting Position (*x*_0_*, y*_0_) [mm, mm]	*a_start_* [deg]	Attempt	*x_ref_* [mm]	*y_ref_* [mm]	*a_end_* [deg]	*Error x_ref_* [mm]	*Error y_ref_* [mm]	*Error a_end_* [deg]
SP1 (600, 2450)	−30	1	521	318	15	−171	32	−15
		2	591	425	25	−241	−75	−25
		3	584	331	18	−234	19	−18
	0	1	507	422	24	−157	−72	−24
		2	560	536	0	−210	−186	0
		3	511	467	8	−161	−117	−8
	30	1	502	467	−39	−152	−117	39
		2	453	496	−63	−103	−146	63
		3	470	502	−49	−120	−152	49
SP2 (900, 2450)	−30	1	575	579	16	−225	−229	−16
		2	554	444	9	−204	−94	−9
		3	556	680	16	−206	−330	−16
	0	1	477	502	30	−127	−152	−30
		2	471	469	6	−121	−119	−6
		3	468	434	11	−118	−84	−11
	30	1	545	570	−58	−195	−220	58
		2	544	552	−61	−194	−202	61
		3	377	414	−61	−27	−64	61
SP3 (1200, 2450)	−30	1	585	530	5	−235	−180	−5
		2	555	694	7	−205	−344	−7
		3	538	622	2	−188	−272	−2
	0	1	358	360	−59	−8	−10	59
		2	432	385	−65	−82	−35	65
		3	428	467	−69	−78	−117	69
	30	1	311	291	−74	40	59	74
		2	138	127	−81	212	223	81
		3	313	335	−74	38	15	74
SP4 (1500, 2450)	−30	1	438	397	−21	−88	−47	21
		2	539	529	−4	−189	−179	4
		3	492	474	−15	−142	−124	15
	0	1	313	356	−63	37	−6	63
		2	281	237	−63	69	113	63
		3	384	408	−62	−34	−58	62
	30	1	481	505	−75	−131	−155	75
		2	333	373	−87	17	−23	87
		3	436	433	−78	−86	−83	78

**Table 7:** Mean Error and Standard Deviation of Error for Fuzzy Logic Algorithm.

Starting Position (*x*_0_*, y*_0_) [mm, mm]	*a_start_* [deg]	Mean *Error x_ref_* [mm]	Mean *Error y_ref_* [mm]	Mean *Error a_end_* [deg]	Std. Deviation of *Error x_ref_* [mm]	Std. Deviation of *Error y_ref_* [mm]	Std. Deviation of *Error a_end_* [deg]
SP1 (600, 2450)	−30	46	27	7	27	14	3
	0	31	24	5	22	11	1
	30	17	36	2	15	3	1
SP2 (900, 2450)	−30	90	16	11	79	10	2
	0	73	23	8	29	6	1
	30	29	83	3	29	96	1
SP3 (1200, 2450)	−30	53	13	9	29	3	2
	0	42	30	7	13	2	2
	30	34	46	4	9	46	4
SP4 (1500, 2450)	−30	48	67	9	15	87	2
	0	37	27	4	16	5	1
	30	59	82	2	29	105	2

**Table 8:** Mean Error and Standard Deviation of Error for Dead Reckoning Algorithm.

Starting Position (*x*_0_*, y*_0_) [mm, mm]	*a_start_* [deg]	Mean *Error x_ref_*	Mean *Error y_ref_*	Mean *Error a_end_*	Std. Deviation of *Error x_ref_*	Std. Deviation of *Error y_ref_*	Std. Deviation of *Error a_end_*
SP1 (600, 2450)	−30	215	42	19	31	47	4
	0	176	125	11	24	47	10
	30	125	138	50	20	15	10
SP2 (900, 2450)	−30	211	217	14	9	96	3
	0	122	118	15	4	28	10
	30	138	162	60	79	69	1
SP3 (1200, 2450)	−30	209	265	5	20	67	2
	0	56	54	65	34	46	4
	30	96	99	77	82	89	3
SP4 (1500, 2450)	−30	139	117	13	41	54	7
	0	47	59	62	43	71	0
	30	78	87	80	62	54	5

**Table 9:** Comparison of average error value and standard deviation for all tests (fuzzy controller–bold values).

	XREF [mm]		YREF [mm]		αend [deg]	
Mean value of error	47	134	39	124	6	39
Std. Deviation of error	52	102	59	114	4.48	38.9

## Data Availability

The original contributions presented in this study are included in the article material. Further inquiries can be directed to the corresponding author.
